# Endothelial-dependent dilation following chronic hypoxia involves TRPV4-mediated activation of endothelial BK channels

**DOI:** 10.1007/s00424-018-2112-5

**Published:** 2018-01-29

**Authors:** Jay S. Naik, Benjimen R. Walker

**Affiliations:** Department of Cell Biology and Physiology, University of New Mexico, MSC08 4750, Albuquerque, NM 87131 USA

**Keywords:** TRPV4, Endothelium, BK channel, Chronic hypoxia

## Abstract

Following chronic hypoxia (CH), the systemic vasculature exhibits blunted vasoconstriction due to endothelial-dependent hyperpolarization (EDH). Previous data demonstrate that subsequent to CH, EDH-mediated vasodilation switches from a reliance on SK_ca_ and IK_ca_ channels to activation of the endothelial BK_ca_ channels (eBK). The mechanism by which endothelial cell stimulation activates eBK channels following CH is not known. We hypothesized that following CH, EDH-dependent vasodilation involves a TRPV4-dependent activation of eBK channels. ACh induced concentration-dependent dilation in pressurized gracilis arteries from both normoxic and CH rats. Inhibition of TRPV4 (RN-1734) attenuated the ACh response in arteries from CH rats but had no effect in normoxic animals. In the presence of L-NNA and indomethacin, TRPV4 blockade attenuated ACh-induced vasodilation in arteries from CH rats. ACh elicited endothelial TRPV4-mediated Ca^2+^ events in arteries from both groups. GSK1016790A (GSK101, TRPV4 agonist) elicited vasodilation in arteries from normoxic and CH rats. In arteries from normoxic animals, TRAM-34/apamin abolished the dilation to TRPV4 activation, whereas luminal iberiotoxin had no effect. In CH rats, only administration of all three K_ca_ channel inhibitors abolished the dilation to TRPV4 activation. Using Duolink®, we observed co-localization between Cav-1, TRPV4, and BK channels in gracilis arteries and in RAECs. Disruption of endothelial caveolae with methyl-β-cyclodextrin significantly decreased ACh-induced vasodilation in arteries from both groups. In gracilis arteries, endothelial membrane cholesterol was significantly decreased following 48 h of CH. In conclusion, CH results in a functional coupling between muscarinic receptors, TRPV4 and K_ca_ channels in gracilis arteries.

## Introduction

Chronic hypoxia (CH) is associated with pathological conditions that result in hypoxemia, as well as from prolonged residence at high altitude. We have previously demonstrated that agonist-induced vasoconstriction [8, 13, 26] and myogenic reactivity [7, 22] are attenuated within the systemic circulation following CH. These diminished responses to vasoconstrictor stimuli are endothelium-dependent [5, 13] and associated with endothelial cell membrane potential hyperpolarization [33]. Since these prior experiments were performed under normoxic conditions, this diminished vasoconstrictor reactivity appears to be an adaptation to prolonged hypoxic exposure, rather than an effect of acute hypoxia per se. We have further shown that this reduction in vasoconstrictor reactivity following CH appears to involve the presence of functional endothelial large conductance Ca^2+^-activated K^+^ channels (eBK_ca_). The present study continues this work by determining the mechanism of eBK channel activation.

In the majority of the systemic vasculature, if expressed, eBK_ca_ on endothelial cells are electrically quiescent, but may be disinhibited in a pathophysiological setting [36]. We have previously demonstrated that following CH, eBK_ca_ channels are involved in endothelial and smooth muscle cell hyperpolarization and endothelium-dependent vasodilation [13, 33, 34]. The unmasking of functionally active BK channels within the endothelium following CH appears to be secondary to a CH-induced reduction in endothelial membrane cholesterol content [27, 33, 34]. Indeed, BK channels have been shown to be directly regulated by membrane cholesterol through a protein-sterol interaction [1, 2]. Although our previous work provides evidence that endothelial cells express functional BK channels following CH, the mechanism(s) by which eBK_ca_ channels are activated within the endothelium in response to G-protein receptor stimulation has not been determined.

Within the resistance vasculature, nitric oxide- and prostacyclin-independent endothelial hyperpolarization (EDH) and the subsequent vasodilation are dependent on activation of intermediate (IK_ca_) and small (SK_ca_) conductance Ca^2+^-activated K^+^ channels [10]. Activation of these channels is thought to be mediated by the influx of extracellular Ca^2+^ through transient receptor potential cation channel V4 (TRPV4) and through inositol trisphosphate receptors in the endoplasmic reticulum in a manner that is comparable to the relationship between BK_ca_ channels and ryanodine-sensitive channels (i.e., Ca^2+^ sparks) in vascular smooth muscle cells [39, 40]. Activation of endothelial TRPV4 channels results in spatially discrete, localized increases in Ca^2+^, referred to as TRPV4 sparklets [39]. Previous work has demonstrated that TRPV4 channels form a functional unit with K_ca_ channels within caveolae of endothelial cells. Indeed, TRPV4 and SK_ca_, but not IK_ca_, have been shown to be localized within discrete signaling domains within caveolae [11]. Our laboratory as well as others has shown that EDH-dependent signaling involves IK_ca_ and SK_ca_ channel stimulation in arteries from normoxic animals [9, 13]. However, in arteries from CH animals, EDH dilation predominately involves eBK_ca_ channel activation [13]. It is unknown if eBK_ca_ channels are regulated by localized Ca^2+^ release events similar to IK_Ca_ and SK_Ca_ channels. Thus, we hypothesized that following CH, EDH-induced vasodilation involves TRPV4-dependent activation of eBK_ca_ channels localized within caveolae.

## Materials and methods

### Ethical approval

Male Sprague-Dawley rats (Envigo, 200–250 g) were used for all experiments. Rats were euthanized with a lethal concentration of pentobarbital sodium (200 mg/kg ip), and aorta or gracilis arteries were collected. The Institutional Animal Care and Use Committee of the University of New Mexico School of Medicine reviewed and approved all animal protocols. All protocols conformed to National Institutes of Health guidelines for animal use.

### Hypoxia exposure protocol

A subset of rats studied were exposed to CH for 48 h in a hypobaric chamber maintained at 380 Torr. We have previously demonstrated that 48 h of CH is sufficient to elicit an endothelial-dependent blunting of vasoconstrictor reactivity that is similar to what we have observed following a 4-week exposure [14, 26]. Normoxic animals were maintained in the same facility under normobaric conditions and housed in identical cages under the same light:dark cycle.

### Isolated gracilis artery preparation

Gracilis artery segments (~ 200 μm, i.d.) were isolated in physiological salt solution [PSS; containing (in mM) 129.8 NaCl, 5.4 KCL, 0.83 MgSO_4_, 0.43 NaH_2_PO_4_, 19 NAHCO_3_, 1.8 CaCl_2_, and 5.5 glucose], gassed with 21% O_2_-6% CO_2_-balance N_2_. Artery segments were secured to glass cannulas and then pressurized to 80 mmHg in a vessel chamber (Living System Instrumentation). Endothelium-intact arteries were superfused at a rate of 5 ml/min with PSS heated to 37 °C and gassed with (21% O_2_-6% CO_2_-balance N_2_). A subset of arteries underwent endothelial disruption by rubbing the lumen of the artery with a strand of moose mane inserted into the free distal end of the vessel as previously described [16]. In these arteries, dislodged endothelial cells were flushed from the artery before the distal end was cannulated and secured with silk sutures. In addition, some arteries were pretreated intralumminally with the caveolae disrupting agent methyl-β-cyclodextrin (MbCD; 10 mM) for 30 min prior to ACh-induced dilation experiments [19].

### Vasodilation studies

Arterial inner diameter was recorded in cannulated, pressurized arteries using edge detection software (IonOptix). Arteries were equilibrated at 37 °C in warmed, oxygenated PSS for 30 min prior to the start of the experiment. Although gracilis arteries develop myogenic tone, we have previously observed reduced myogenic tone following CH. Thus, to insure similar levels of basal tone, following equilibration, arteries were pre-constricted to ~ 50% of their initial resting diameter using phenylephrine (PE) and vasodilation was measured during cumulative addition of the endothelium-dependent dilator acetylcholine (ACh) or the TRPV4 agonist, N-((1S)-1-[4-((2S)-2-[(2,4-Dichlorophenyl)sulfonyl]amino-3-hydroxypropanoyl)-1-piperazinyl]carbonyl-3-methylbutyl)-1-benzothiophene-2-carboxamide (GSK1016790A, Sigma-Aldrich). In subsets of experiments, artery segments were pretreated in the lumen and bath with the TRPV4 channel antagonist, RN-1734 (30 μM), N-nitro-L-arginine (100 μM, L-NNA) and indomethacin (10 μM), TRAM-34 (1 μM), and apamin (100 nM). To inhibit selectively endothelial BK channels, iberiotoxin (100 nM, IBTx) was only administered luminally. The percent dilation in response to ACh or GSK101 was calculated as ((dilation diameter − PE diameter) ∕ (Ca^2+^-free diameter − PE diameter)) × 100.

### Immunohistochemistry studies

Protein localization was performed in tissue sections of gracilis muscle from control rats. Briefly, gracilis muscles were fixed with 2% paraformaldehyde and embedded in paraffin for tissue sectioning. Five-micron-thick sections were blocked with 5% normal donkey serum and incubated overnight with mouse anti-BK (1:200, Abcam [ab99046]), rabbit anti-TRPV4 (1:100, Abcam [ab39260]), or goat anti-caveolin-1 (Cav-1; 1:200, Abcam [ab192452]). All sections were treated with mouse or goat anti-alpha actin (1:300). Tissue sections were then incubated with appropriate secondary antibodies for 20 min at RT. 633 hydrazide (1 μm) was used to stain the internal elastic lamina. Samples were mounted with anti-fade mounting media and images were acquired using a confocal microscope (TCS SP5; Leica Microsystems).

### Proximity ligation assay

Protein-protein co-localization in gracilis artery was determined using Duolink in situ proximity ligation assay (PLA) according to the manufacturer’s instructions as previously described [38]. Briefly, whole gracilis muscles were fixed with 2% paraformaldehyde and embedded in paraffin for tissue sectioning. Five-micron-thick sections were incubated with Duolink blocking buffer for 30 min at 37 °C, then incubated overnight with mouse anti-BK (1:200, Abcam [ab99046]) and rabbit anti-TRPV4 (1:100, Abcam [ab39260]) or each with goat anti-caveolin-1 (Cav-1; 1:200, Abcam [ab192452]) in separate tissue sections. Tissue sections were then incubated with the appropriate PLA probes (1:5) for 1 h at 37 °C. SYTOX Green (1:5000) was used as a nuclear stain. 633 hydrazide (1 μm) was used to stain the internal elastic lamina. Proximity ligation assays examining the same protein pairs were also performed in rat aortic endothelial cells (Cell Applications, Inc.). Samples were mounted with Duolink mounting media and z-stack images of the PLA interactions were acquired using a confocal microscope (TCS SP5; Leica Microsystems). Negative controls were completed by (1) omission of primary antibody (not shown) and (2) incubation of each primary antibody individually with both PLA probes. Quantification of the number of dots/endothelial cell nuclei was performed by an individual blinded to treatment and antibody pair.

### Endothelial Ca^2+^ events

Basal and ACh-induced endothelial Ca^2+^ events were recorded in cannulated, pressurized arteries in the presence of L-NNA (100 μM) and indomethacin (10 μM). Briefly, the lumen of the artery was then filled with a HEPES-buffered PSS solution containing fluo-4 (5 μM), Oregon green 488 BAPTA-1 (5 μM), and .025% Pluronic F-127 and allowed to incubate for 15 min at RT and then flushed with buffer for an additional 15 min. We did not observe smooth muscle cells when the focal plane was moved across the vascular wall, confirming selective loading of endothelial cells with the fluorophore. Arteries were then equilibrated at 37 °C in warmed, oxygenated HEPES-PSS for 15 min prior to the start of the experiment. Arteries were pretreated with vehicle or the TRPV4 antagonist GSK2193874 (300 nM, GSK219 Sigma-Aldrich) for at least 30 min prior to experimentation. TRPV4-dependent Ca^2+^ events were assessed by exciting the fluorophores with a solid-state 488-laser and emissions > 500 nm were collected using an Olympus IX71 microscope with a × 40 oil-immersion objective, an Andor camera, and a spinning disk confocal scanning unit (CSU22, Yokogawa Electrical Corp.). Images were collected under baseline and following 1 μM ACh. Six hundred images were collected at a frame rate of 50–60 Hz. Diverse Ca^2+^ events within the endothelium were quantified using ImageJ plugin LC_Pro (10-pixel diameter region of interest and a *P* < 0.05 level of significance), a detection, and analysis algorithm that automatically assigns ROI by detecting fluorescence signals that are statistically significant above background and reports the spatial and temporal parameters of these events. As a result, detectable Ca^2+^ events whose fluorescent intensity is statistically significant are reported. This eliminates the need for the investigator to identify ROIs and determine inclusion/exclusion criteria for individual Ca^2+^ events based on arbitrary spatiotemporal event parameters. Since the majority of events were small spatially discrete events (i.e., 10 μm^2^), only events that were less than 10 μm^2^ were included in the statistical analysis of total Ca^2+^ events (Fig. 9e).

### Endothelial membrane cholesterol

Rat aorta was cleaned of perivascular fat and cut open longitudinally. Strips of aorta were placed in EBM-2 media containing 10% fetal bovine serum. Endothelial cell sheets were obtained by incubating strips of aorta with 0.2 mg/ml DTT, 480 U/ml collagenase II, and 7 U/ml displace for 45 min and 37 °C. Artery strips were then placed in HEPES-bufferd physiological saline solution containing 0.1% bovine serum albumin. Endothelial cell sheets were then released by gentle trituration with a small-bore fire-polished Pasteur pipette. Endothelial cell sheets were allowed to adhere to poly-l-lysine coated coverslips and were treated with 4% paraformaldehyde for 15 min at room temperature and then washed three times with PBS. Membrane cholesterol was detected by incubating cells with the fluorescent cholesterol marker filipin III (Sigma, 75 μg/ml) for 90 min at room temperature under light-protected conditions. Coverslips were mounted on Superfrost microscope slides using anti-fade mounting media. The samples were imaged by fluorescence confocal microscopy (Zeiss LSM 510 AxioObserver; Göttingen, Germany) using a 405-nm laser (excitation), a 420-nm-long pass filter (emission), and a Plan-Neofluor × 40/1.3 oil objective. Filipin staining was quantified using NIH Image J. Fluorescence intensity was quantified by setting a threshold using blank control (filipin-untreated group). Thirty-five to forty sheets per animal were analyzed. Fluorescence of each aortic endothelial cell sheet was calculated and averaged to determine mean fluorescence for each animal.

### Statistical analysis

Data are presented as mean ± SEM and were analyzed using a one-way ANOVA or two-way ANOVA, as appropriate. Where significant main effects occurred, pairwise comparisons were performed using Tukey’s or Bonferroni’s post hoc tests (Graphpad Prism). *P* < 0.05 was considered statistically significant for all analyses.

### Reagents

RN-1734, TRAM-34, iberiotoxin, and apamin were purchased from Tocris Biosciences. Sytox Green was purchased from ThemoFisher Scientific. Duolink in situ proximity ligation assay and all other reagents were purchased from Sigma-Aldrich.

## Results

### Endothelial membrane cholesterol

**Fig. 1:**
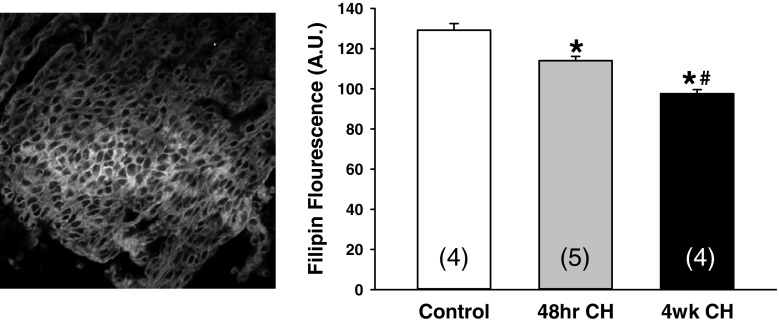
Endothelial membrane cholesterol was quantified using filipin staining in aortic endothelial cell sheets from normoxic controls and animals exposed to either 48 h or 4 weeks of CH. Sample size is indicated by the number in parentheses. *Different from normoxia (*P* < 0.05). ^#^Different from 48 h CH (*P* < 0.05)

Endothelial membrane cholesterol was significantly decreased following 48 h of CH compared to endothelial sheets from normoxic controls. Exposing animals to 4 weeks of CH resulted in a further reduction in cholesterol levels (Fig. 1). These results demonstrate that CH exposure results in an exposure duration-dependent reduction in membrane cholesterol and that in as early as 48 h of exposure, there is a significant reduction in endothelial cholesterol levels. These results support our previous work demonstrating a membrane cholesterol-dependent appearance of functional eBK channels in the endothelium of gracilis arteries following 48 h of CH [33].

### Role of caveolae in ACh-induced vasodilation

**Fig. 2:**
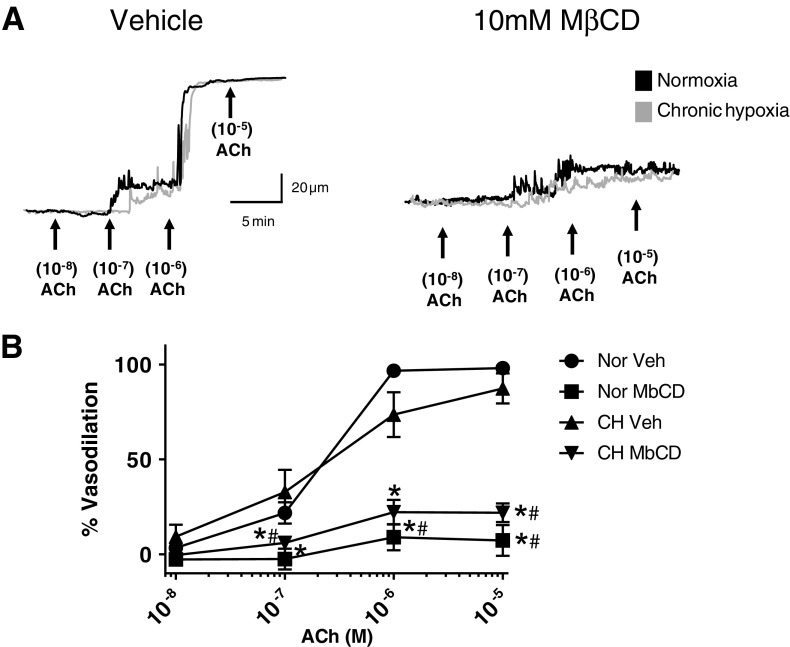
Role of caveolae in Ach-induced vasodilation was assessed in gracilis arteries from normoxic and chronic hypoxic (CH) rats. Arteries were pretreated with N-nitro-L-arginine (100 μM, L-NNA) and indomethacin (10 μM, INDO). A subset of arteries from both groups was treated intraluminally with 10 mM methyl-β-cyclodextrin (MbCD) for 30 min. Representative traces of the ACh-induced vasodilation in gracilis arteries from normoxic and CH rats pretreated with either vehicle or luminal MβCD (**a**). *N* = 5/group. *Different from Veh within groups (*P* < 0.05). ^#^Different from Veh between groups

Administration of ACh in the presence of L-NNA and indomethacin elicited a concentration-dependent dilation in arteries from both groups. Disruption of endothelial cell caveolae structure using methyl-β-cyclcodextrin attenuated the EDH-dependent dilation similarly in arteries from both groups (Fig. 2). These results suggest that localization of the components of muscarinic receptor signaling within caveolae is required for ACh-induced vasodilation. Vasodilation in response to the endothelial-independent dilator sodium nitroprusside (0.1 μM) was unaltered by luminal MbCD treatment (96.3 ± 6% and 99.3 ± 1.1, for vehicle and MbCD treated, respectively, *n* = 3/group).

### Role of TRPV4 in endothelial-dependent dilation in arteries from normoxic and CH rats

**Fig. 3:**
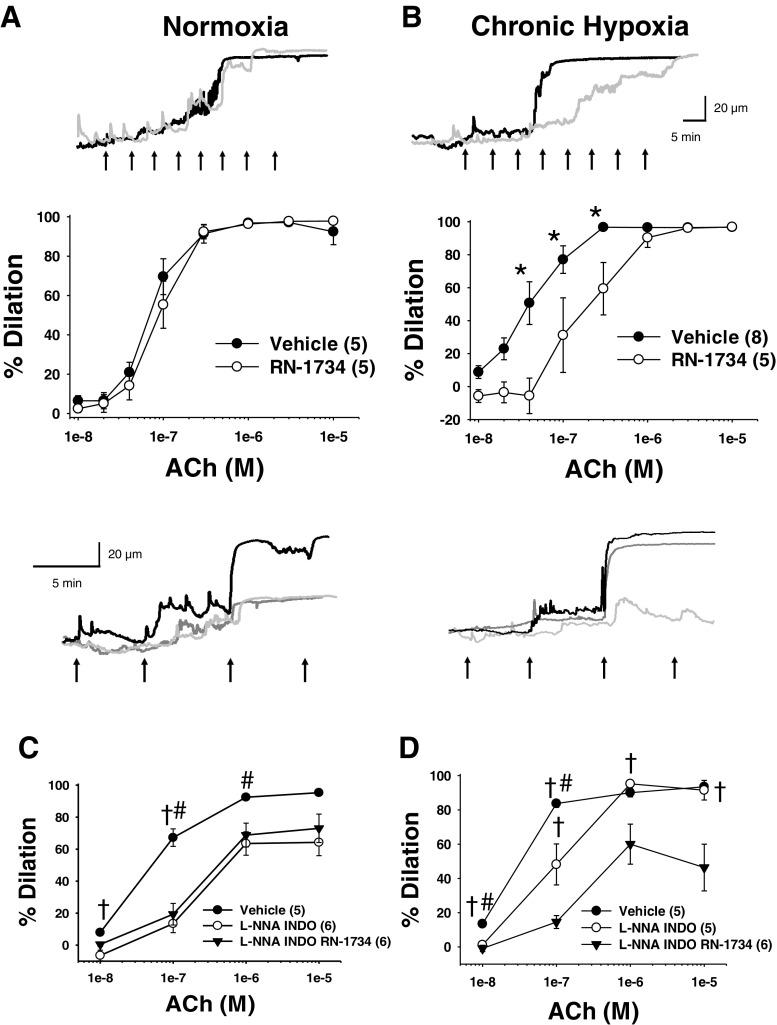
Representative traces of aceylcholine (ACh)-induced vasodilation in the presence of vehicle (black lines), RN-1734 (gray lines), or L-NNA and indomethacin (dark gray lines) in gracilis arteries from normoxic and CH rats. ACh-induced dilation was assessed in arteries from normoxic and CH rats plus and minus the TRPV4 antagonist, RN-1734 (30 μM) under control conditions [**a** and **b**] or in the presence of N-nitro-L-arginine (100 μM, L-NNA) and indomethacin (10 μM, INDO) [**c** and **d**]. Sample size is indicated by the number in parentheses. ^↑^Indicates approximate time point for increasing concentrations of ACh administration. *Different from RN-1734 (*P* < 0.05). ^†^Different from L-NNA/INDO/RN-1734 (*P* < 0.05). ^#^Different from L-NNA/INDO (*P* < 0.05)

To determine the role of endothelial TRPV4 in endothelium-dependent vasodilation, we examined the response to ACh in the presence and absence of RN-1734 in arteries from normoxic and CH rats. ACh produced a concentration-dependent vasodilation in arteries from both normoxic (Fig. 3a, 1 × 10^−8^–1 × 10^−5^) and CH animals (Fig. 3b). Unexpectedly, pretreatment of arteries with RN-1734 did not alter ACh-induced vasodilation in arteries from normoxic animals (Fig. 3a). Whereas, TRPV4 inhibition attenuated ACh vasodilation in arteries from CH rats (Fig. 3b). The EC_50_ values were 66 and 73 nM for normoxic vehicle and normoxic RN-1734, respectively. In arteries from CH rats, the EC_50_ was significantly different between CH vehicle and CH RN-1734 (50 and 123 nM, respectively). ACh-induced dilation was not significantly different in arteries from normoxic and CH rats.

EDH-mediated vasodilation involves activation of endothelial SK_ca_ and IK_ca_ channels [45]. Furthermore, SK_ca_ and IK_ca_ channels are involved in TRPV4-induced vasodilation [39]. Thus, arteries were pretreated with L-NNA and indomethacin to isolate the EDH component of ACh-induced vasodilation. In arteries from normoxic animals (Fig. 3c), inhibition of nitric oxide synthase and cyclooxygenase attenuated ACh-induced dilation and the addition of the TRPV4 inhibitor (RN-1734) did not further alter the response. In contrast, inhibition of nitric oxide synthase and cyclooxygenase did not significantly alter ACh dilation in arteries from CH animals (Fig. 3d), whereas inhibition of TRPV4 attenuated vasodilation. Taken together, these results suggest a loss of nitric oxide/cycloxygenase component of ACh-induced vasodilation occurs in arteries from CH rats. Furthermore, CH exposure couples muscarinic receptor signaling to TRPV4 activation.

### GSK1016790A-induced vasodilation

**Fig. 4:**
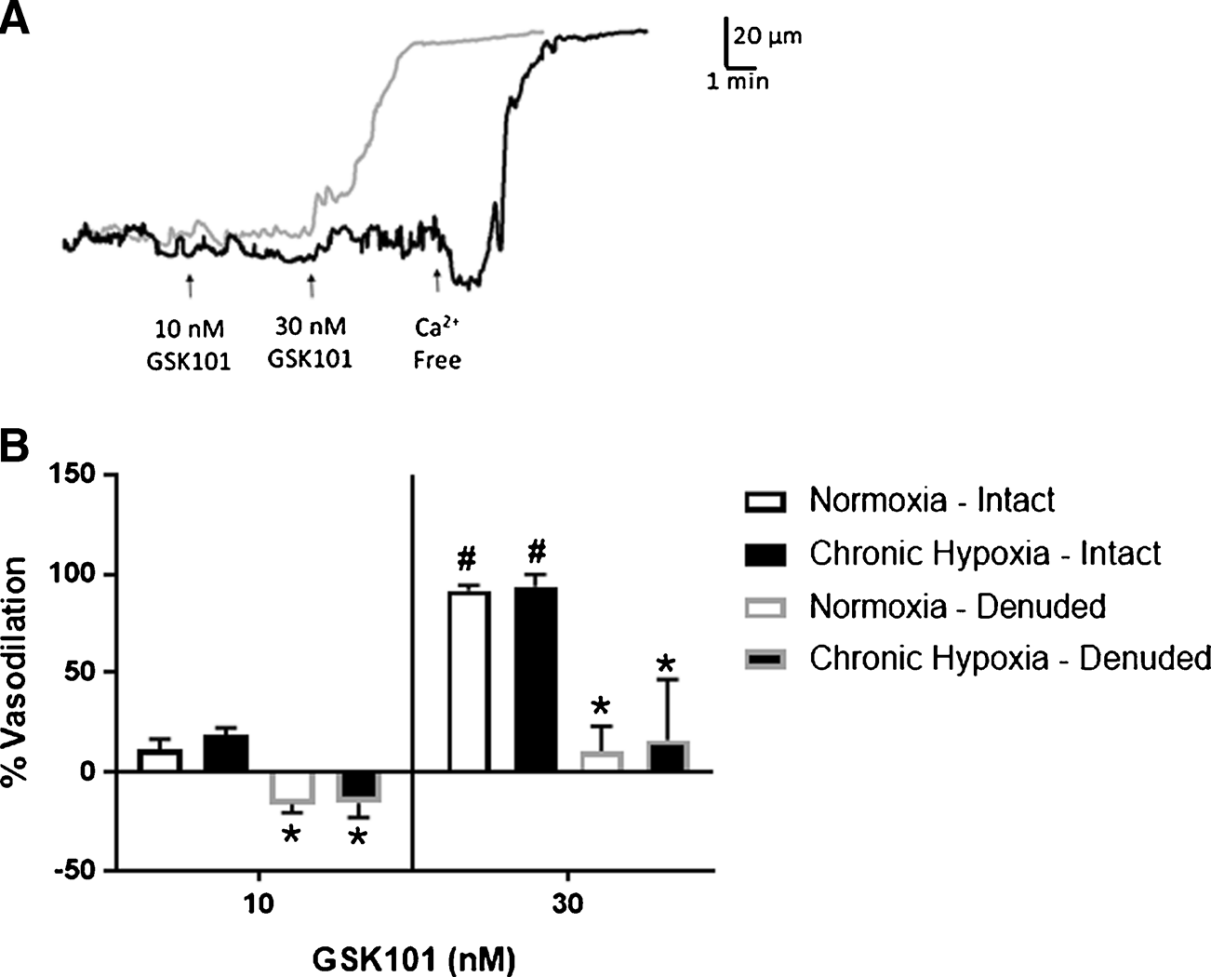
TRPV4-dependent vasodilation in gracilis arteries. Representative traces of GSK101-induced vasodilation in endothelium intact (gray lines) and denuded (black lines) gracilis arteries from normoxic rats [**a**]. Summary of the GSK1016790A (GSK101)-induced dilation under endothelium intact and denuded conditions [**b**]. GSK101 elicits a concentration-dependent vasodilation in arteries from normoxic and CH rats. TRPV4-dependent vasodilation was inhibited by disruption of the endothelium in arteries from both groups. *n* = 5/group. *Different from endothelium intact within groups (*P* < 0.05). ^#^Different from 10 nM GSK101 within groups (*P* < 0.05)

TRPV4 channels are expressed on both vascular smooth muscle and endothelial cells. In arteries from both normoxic control and CH rats, administration of the TRPV4 agonist GSK101 elicited a concentration-dependent dilation that was inhibited by disruption of the endothelium, demonstrating the endothelium dependency of the TRPV4-mediated dilation. (Fig. 4). Pretreating arteries with the TRPV4 antagonist RN-1734 abolished the vasodilation in response to 30 nM GSK101 (− 7 ± 4 and − 15 ± 1% for normoxic and CH groups, respectively). There are two possibilities to account for the vasoconstriction that occurred during the administration of the lower concentration of TRPV4 agonist in arteries pretreated with a TRPV4 antagonist. (1) The arteries developed more myogenic tone during the agonist treatment period, in the absence of a dilator signal, and (2) since it appears that dilation is response to TRPV4 activation is endothelial-dependent, it is possible that activation of smooth muscle TRPV4 increases [Ca^2+^]_*i*_, resulting in contraction. Indeed, contraction in response to TRPV4 activation has been shown in airway smooth muscle [21].

### Role of K_ca_ channels in TRPV4-induced dilation in arteries from normoxic and CH animals

**Fig. 5:**
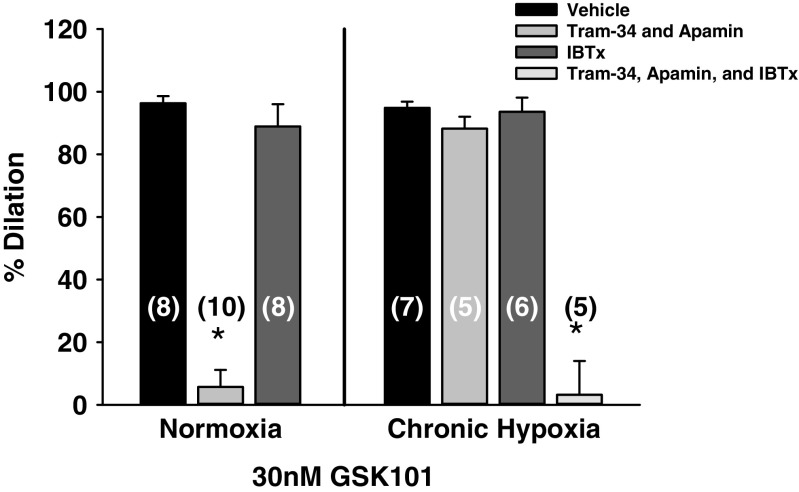
Role of K_ca_ channels in TRPV4-mediated vasodilation was assessed in gracilis arteries from normoxic and CH rats. Arteries were pretreated with N-nitro-L-arginine (100 μM, L-NNA) and indomethacin (10 μM, INDO). GSK1016790A (GSK101)-induced dilation was examined in artery segments pretreated with either vehicle, the BK channel inhibitor, iberiotoxin administered in the lumen (100 nM, IBTx), the IK and SK channel blockers, TRAM-34 (1 μM) and apamin (100 nM), respectively, or the combination of all three K_ca_ channel inhibitors. Sample size is indicated by the number in parentheses. *Different than vehicle

In the presence of vehicle, administration of the TRPV4 agonist GSK101 elicited dilation in arteries from normoxic and CH rats, which were not significantly different (Fig. 5). In arteries from normoxic animals, inhibition of SK_ca_ and IK_ca_ channels abolished the dilation in response to TRPV4 activation. In addition, in arteries from normoxic animals, GSK101-induced vasodilation was not different from vehicle conditions in the presence of eBK_ca_ channel inhibition. However, in arteries from CH rats, inhibition of SK_ca_ and IK_ca_ or eBK_ca_ channels separately failed to alter GSK101-induced vasodilation. However, administration of all three K_ca_ channel inhibitors abolished this response. These results suggest that TRPV4 channels are expressed on the endothelium of arteries from both normoxic and CH rats. In addition, there appears to be a switch from a phenotype where endothelial-dependent dilation relies on Ca^2+^-activated SK_ca_ and IK_ca_ channels to a pathway that incorporates the involvement of eBK_ca_ channels in arteries from animals exposed to CH.

### Expression of TRPV4, BK channels, and Cav-1 in gracilis muscle

**Fig. 6:**
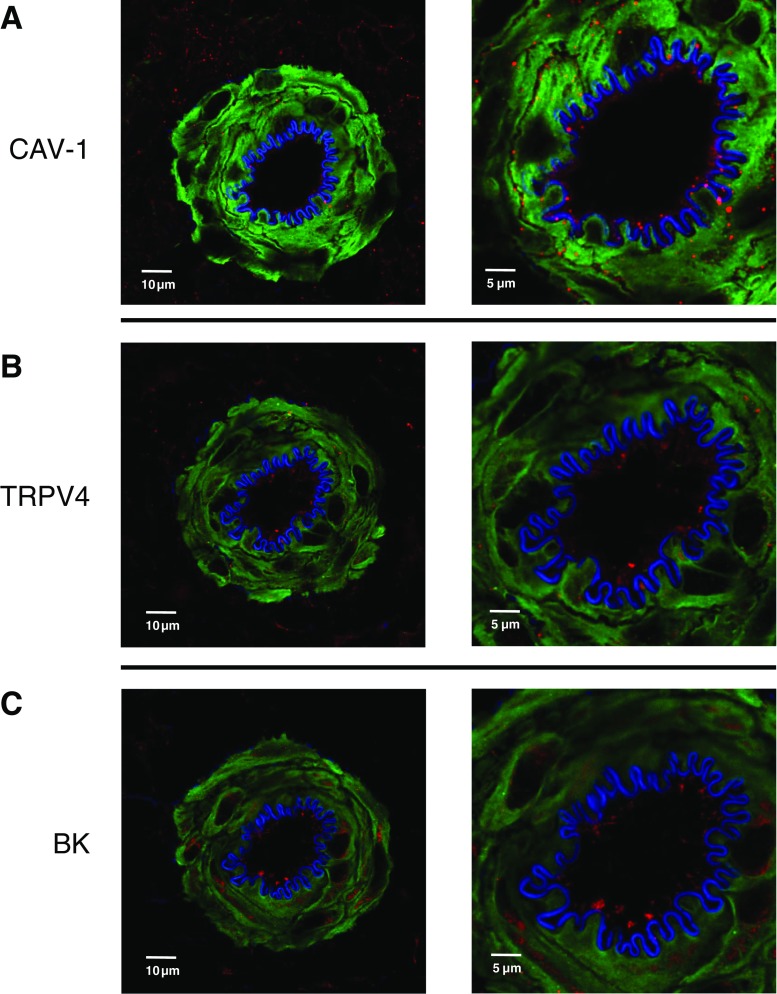
Representative confocal images of the protein expression of Cav-1 [**a**], TRPV4 [**b**], and BK channels [**c**] in gracilis arteries of control rats. Alpha actin (green), protein of interest (red), and internal elastic lamina (blue)

We examined the expression of TRPV4, BK channels, and Cav-1 in sections of gracilis artery from control rats using immunofluorescence. Cav-1 expression was observed in both skeletal and smooth muscles as well as the endothelium of gracilis arteries (Fig. 6a). Whereas, TRPV4 and BK channels were expressed in both the endothelial and medial layers of gracilis arteries (Fig. 6b, c).

### Co-localization of TRPV4, BK channels, and Cav-1

**Fig. 7:**
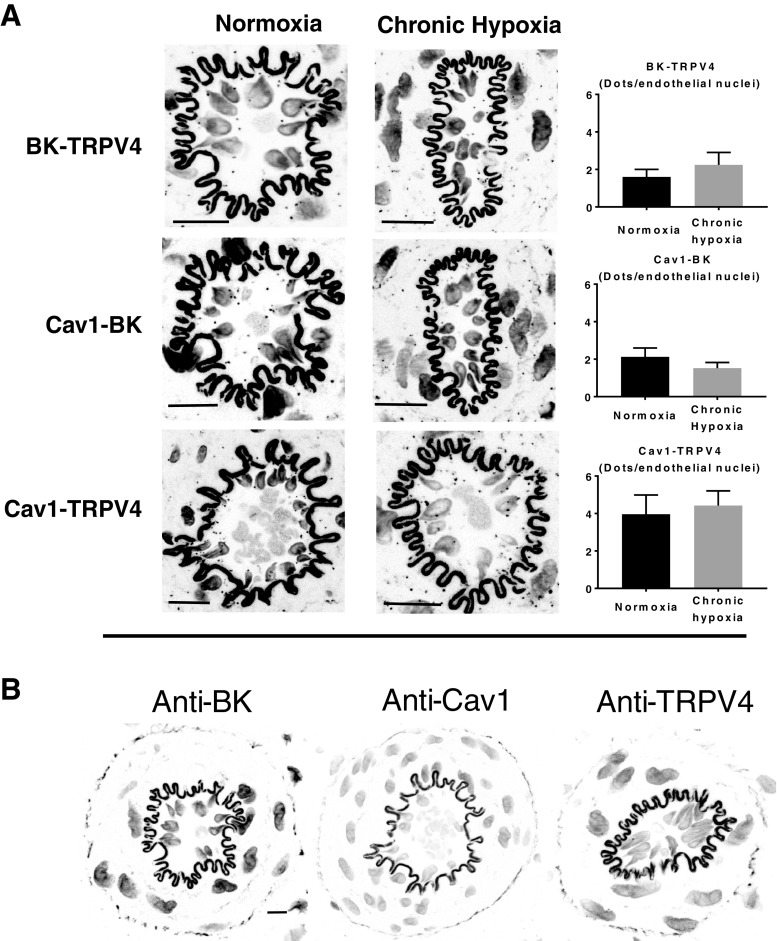
Co-localization of TRPV4, eBK channels, and Cav-1 in the endothelium of gracilis arteries from normoxic controls and CH rats. **a** Representative confocal images of the Duolink PLA interactions (black puncta). The number of puncta within the borders of the internal elastic lamina was quantified and normalized to the number of observed endothelial cell nuclei (*n* = 5 rats/group). For negative controls [**b**], tissue sections were incubated with each primary alone and both PLA probes. Nuclei are labeled with SYTOX. The internal elastic lamina was stained with 633 hydrazide. Bar = 10 μm

**Fig. 8:**
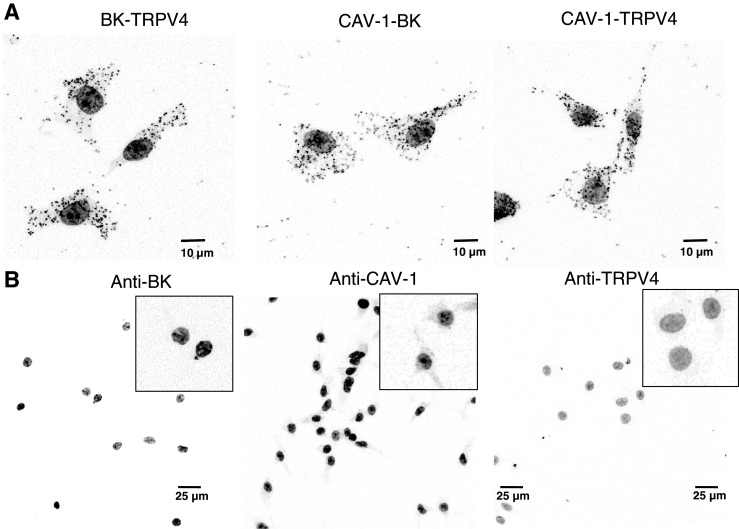
Co-localization of TRPV4, eBK channels, and Cav-1 in RAEC. **a** Representative confocal images of the Duolink PLA interactions (black puncta). For negative controls [**b**], RAEC were incubated with each primary alone and both PLA probes. Nuclei are labeled with SYTOX

TRPV4-induced dilation has been shown to be sensitive to SK_ca_ and IK_ca_ inhibition [39], suggesting these proteins are in close proximity allowing Ca^2+^ entry through TRPV4 channels to serve as a source of activator Ca^2+^ for these channels. The present study demonstrates a role for eBK_ca_ channels in TRPV4-mediated vasodilation in arteries from CH rats. If endothelial TRPV4 serves as a source of activator calcium for eBK_ca_ channels, these proteins must also reside in spatially discrete regions in the plasma membrane. Using Duolink PLA, we observed co-localization of TRPV4 and BK channels in the endothelium of gracilis arteries (Fig. 7a). In addition, both TRPV4 and BK channels co-localized with Cav-1. The degree of spatial localization between any of the protein pairs was not altered by CH exposure (Fig. 7a). In addition, we observed similar spatial localization between TPV4, BK channels, and Cav-1 in RAECs (Fig. 8). As a negative control, either both primary antibodies were omitted or each primary antibody was omitted separately. We failed to observe puncta under these conditions (Figs. 7b and 8b).

### ACh-induced endothelial Ca^2+^ events

**Fig. 9:**
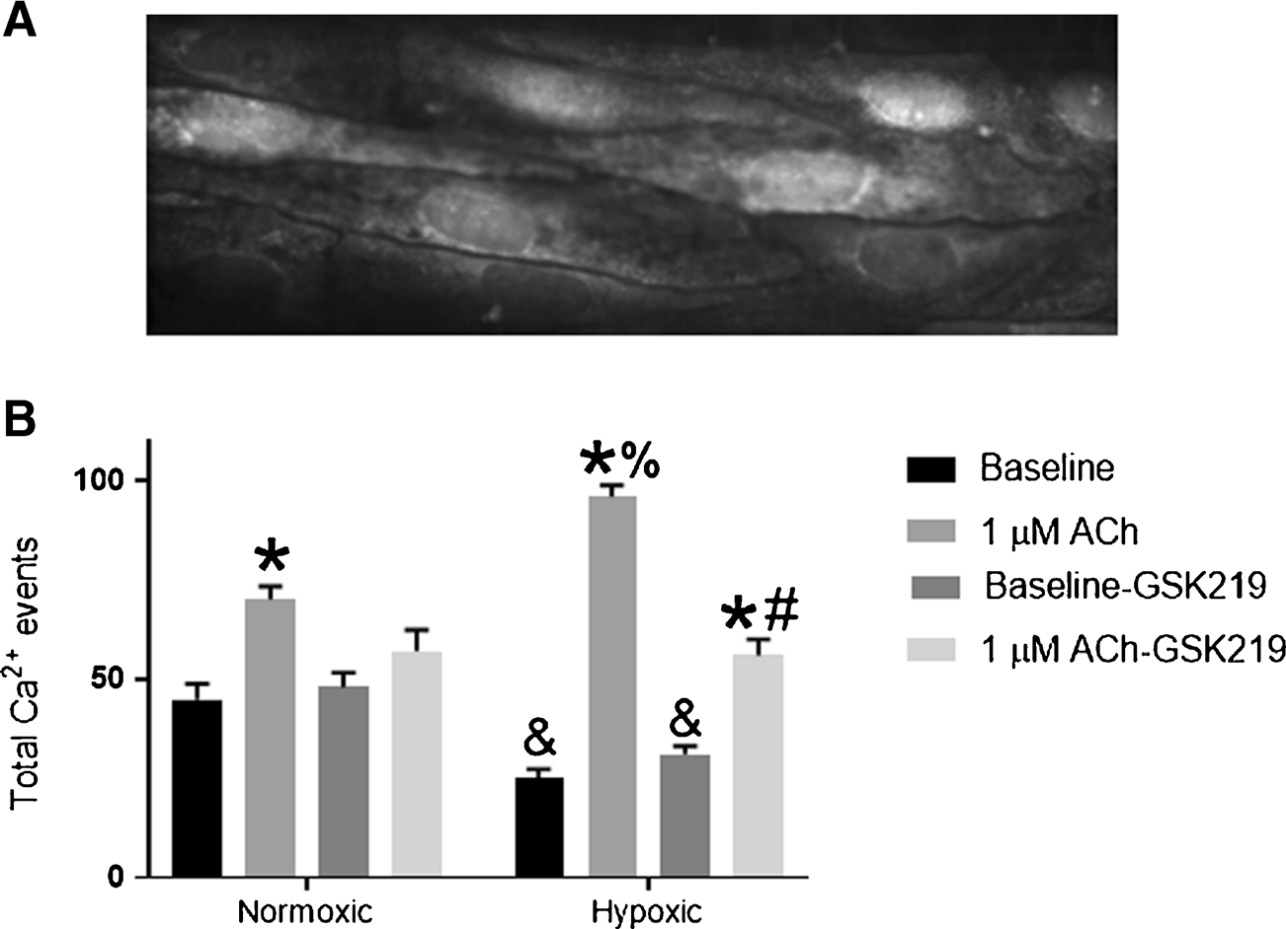
Representative image of a gracilis artery loaded with Ca^2+^ indicators [**a**]. Total basal and ACh-induced Ca^2+^ events [**b**]. Arteries were pretreated with N-nitro-L-arginine (100 μM, L-NNA) and indomethacin (10 μM, INDO). Endothelial cells were selectively loaded with the fluorescent calcium indicators fluo-4 and Oregon Green 488 BAPTA-1. 600 images/artery were collected at 50–60 Hz using a spinning disk confocal microscope (× 40 oil). ACh-induced Ca^2+^ events were assessed in arteries pretreated with vehicle or the TRPV4 antagonist GSK2193874 (300 nM). *Different from baseline or baseline GSK219 within group (*P* < 0.05). ^#^Different from ACh within group (*P* < 0.05). ^&^Different from normoxic baseline and normoxic baseline GSK219 (*P* < 0.05). ^%^Different from normoxic ACh (*P* < 0.05). *N* = 5–6/group

Representative image of the endothelium of a gracilis artery loaded with the Ca^2+^ indicators Fluo-4 and Oregon Green BAPTA-1 (Fig. 9a). The ImageJ plugin LC_Pro captures all calcium events within a pre-determined ROI size that are statistically significant (*P* < 0.05) above background. This approach collects all Ca^2+^ signals within the field of view. Temporal and spatial characteristics of basal and ACh-induced Ca^2+^ events are shown in Table 1. Administration of ACh elicited an increase in total Ca^2+^ events within the endothelium of arteries from both groups (Fig. 9b). ACh induced significantly more Ca^2+^ events in arteries from CH animals compared with normoxic controls, likely the result of TRPV4 activation as the total number of ACh-induced Ca^2+^ events in the presence of GSK219 was not different between control and CH. However, inhibition of TRPV4 channels decreased ACh-induced events in arteries from CH rats. Although there was a trend for ACh to increase the number of total Ca^2+^ events in arteries from control animals in the presence of GSK219, this did not reach statistical significance. There were significantly fewer basal Ca^2+^ events in arteries from CH rats compared to normoxic animals. Inhibition of TRPV4 did not reduce the number of basal Ca^2+^ events in arteries from either group. There are a number of potential ion channels either on the plasma or on ER membrane that could mediate these basal events. This uncertainty makes it difficult to speculate about a mechanism producing the observed decrease in basal activity.

**Table 1:** Temporal and spatial characteristics of basal and ACh-induced Ca^2+^ events

	Frequency (events/site/s)		No. of active sites		Amplitude (*F*/*F*_0_)	
	Baseline	ACh	Baseline	ACh	Baseline	ACh
Normoxia	0.168 ± 0.02	0.159 ± 0.01	5 ± 1	14 ± 3*	1.21 ± 0.04	1.25 ± 0.05
Chronic hypoxia	0.153 ± 0.02	0.182 ± 0.04	4 ± 1	13 ± 1*	1.25 ± 0.05	1.32 ± 0.03

*Significantly different from baseline (*P* < 0.05)

## Discussion

**Fig. 10:**
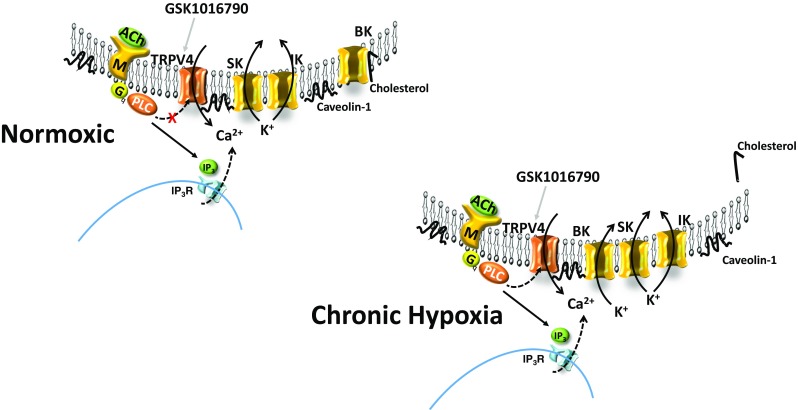
In arteries from normoxic animals, EDHF-dependent dilation does not involve activation of TRPV4 channels. Muscarinic receptor activation does not appear to elicit TRPV4-mediated calcium events in the endothelium of arteries from normoxic animals. However, intact caveolae appear to be required for ACh-induced dilation in arteries from normoxic and CH rats. Direct activation of TRPV4 with GSK1016790A elicits an SK/IK-dependent dilation, suggesting that TRPV4 channels are functionally available in the endothelium. In arteries from animals exposed to CH, endothelial membrane cholesterol is reduced and EDHF-mediated dilation is partially dependent on TRPV4 channels that activate SK, IK, and BK channels. Muscarinic receptor activation increases TRPV4-dependent Ca^2+^ events. Transient receptor potential channel V4 (TRPV4), acetylcholine (ACh), large conductance Ca^2+^-activated K^+^ channel (BK), intermediate conductance Ca^2+^-activated K^+^ channel (IK), small conductance Ca^2+^-activated K^+^ channel (SK), muscarinic receptor (M), inositol trisphosphate (IP_3_), IP_3_ receptor (IP_3_R), endoplasmic reticulum (ER), phospholipase C (PLC). TRPV4 agonist (GSK1016790A)

The present study was designed to investigate whether EDH-dependent vasodilation involves TRPV4-dependent activation of eBK channels following CH. As summarized in Fig. 10, the major findings of the present study are as follows: (1) 48 h of CH reduces endothelial membrane cholesterol; (2) disruption of endothelial caveolae inhibits ACh-induced vasodilation in arteries from normoxic and CH rats; (3) administration of ACh elicits vasodilation that involves activation of TRPV4 channels following CH only; (4) direct pharmacologic activation of TRPV4 elicits endothelium-dependent dilation in rat gracilis arteries; (5) TRPV4-induced dilation is dependent on activation of SK_ca_/IK_ca_ channels in arteries from normoxic animals, but stimulates all three K_ca_ isoforms in CH; and (6) in both gracilis arteries and RAECs, TRPV4 co-localizes with eBK channels and both TRPV4 and eBK co-localize with Cav-1.

The present study provides evidence that the cholesterol content of native aortic endothelial cells is lower after 48 h of CH compared to the endothelium of normoxic controls. It is possible that his reduction in membrane cholesterol following CH results from a decrease in de novo cholesterol synthesis within the endothelium. Hypoxic exposure has been shown to inhibit synthesis of cholesterol. For example, in CHO-7 cells, hypoxic exposure inhibited de novo cholesterol synthesis by stimulating degradation of HMG-CoA reductase [23]. We and others have shown that loss of cholesterol content of the plasma membrane has important physiological implication due to altered ion channel function [2, 33, 43].

Cholesterol-rich membrane regions (caveolae) contain the scaffolding protein, Cav-1 that acts as a scaffolding protein to cluster lipids and signaling molecules within caveolae and may regulate the activity of proteins targeted to caveolae. The results of the present study show that disruption of endothelial caveolae with MβCD largely attenuates EDH-mediated dilation. In addition, our current findings that both TRPV4 and eBK co-localize with Cav-1 provide further evidence for a compartmentalization of TRPV4 and eBK_Ca_ in caveolae of endothelial cells. Indeed, EDH-mediated dilation has been shown to be dependent on intact endothelial caveolae [35]. Moreover, TRPV4 and SK_ca_ channels have been shown to be enriched in caveolae of human microvascular endothelial cells. Mechanical stimulation of these cells via exposure to shear stress led to a co-localization of IK_ca_ channels with Cav-1 and TRPV4 [11]. In the present study, EDH-mediated dilation does not appear to involve activation of TRPV4 channels in arteries from normoxic animals. Although we did not detect a decrease in the association of Cav-1 with TRPV4, the ability of muscarinic receptor signaling to activate TRPV4 channels following CH may result from alterations in membrane cholesterol and/or its association with Cav-1. Indeed, Saliez et al. have shown that expression of Cav-1 is required for EDH-mediated relaxation by altering membrane location and activity of TRPV4 channels in Cav-1 −/− mice [35]. Taken together, our data suggest that a reduction in plasma membrane cholesterol content is required to reveal functional eBK in the endothelium and intact caveolae structure is required for proper muscarinic receptor signaling.

Administration of ACh elicited a concentration-dependent dilation in arteries from both normoxic and CH rats; inhibition of nitric oxide synthase and cyclooxygenase attenuated the response to ACh in arteries from normoxic animals suggesting the presence of both EDH and nitric oxide/prostaglandin components. However, treatment of arteries from CH rats with L-NNA and indomethacin did not alter the response to ACh, suggesting a loss of the nitric oxide/prostaglandin component of the ACh-induced dilation. This is consistent with studies providing evidence that nitric oxide bioavailability is reduced in CH. Although these studies examined the pulmonary circulation, they suggest that nitric oxide bioavailability is reduced due to elevated scavenging by superoxide [15, 25, 37]. Although highly speculative, it is possible that loss of the nitric oxide component of ACh-induced dilation that we observed in gracilis arteries is also mediated by elevated superoxide production.

Inhibition of TRPV4 channels partially blocked ACh-induced dilation and Ca^2+^ events, suggesting a significant contribution of IP_3_R Ca^2+^ release in response to ACh administration [18]. The lack of our ability to detect a significant increase in calcium events in the presence of GSK219 in arteries from control animals may be the result of a type II error, although this is unlikely given the sample size and modest degree of variability. Alternatively, it is possible that ACh can weakly activate TRPV4 channels, which are detected when calcium events are observed directly, but this level of activation is not sufficient to play a significant role in ACh-induced dilation (Fig. 3). Although, muscarinic receptor signaling does not appear to be efficiently coupled to endothelial TRPV4 channels in arteries from normoxic animals, Ca^2+^ influx through TRPV4 channels contributes to ACh-induced dilation in arteries from CH animals. This finding is consistent with Kadowitz et al. who demonstrated in instrumented rats that decreases in mean arterial pressure in response to ACh infusion were not altered by pharmacologic inhibition of TRPV4 channels in control animals [28]. In contrast, others have shown that muscarinic receptor signaling is coupled to TRPV4 activation. For example, blood pressure responses to ACh in global TRPV4 knockout mice were blunted compared to wild-type controls [44]. In studies employing isolated arteries, evidence for TRPV4 involvement in ACh-induced dilation has also been equivocal. For example, ACh-induced dilation in isolated carotid arteries was unaltered in TRPV4 −/− mice compared to wild-type controls [12]. In addition, inhibition of TRPV4 channels blocked GSK101-induced relaxation, but had no effect on either ACh- or sodium nitroprusside-induced responses in rat pulmonary artery rings [41]. In contrast, other findings have supported a role for TRPV4 channels in ACh-dependent dilation. ACh has been shown to elicit an increase in TRPV4-mediated Ca^2+^ events [32, 39, 40], hyperpolarization [6, 20], and endothelial-dependent dilation [6, 20, 39, 40, 42]. The disparity in the involvement of TRPV4-dependent responses in ACh-induced dilation in normal animals maybe due to species differences. Indeed, most of the studies supporting a role for TRPV4 in muscarinic receptor signaling in control animals were performed in mice. It is unlikely that changes in channel expression underlie the appearance of TRPV4 channels in muscarinic receptor signaling given that (1) no differences were observed in degree of co-localization between TRPV4 and either Cav-1 or eBK and (2) direct activation of TRPV4 elicited a similar vasodilation between arteries from control and CH rats. Interestingly, there is emerging evidence suggesting that TRPV channel family members are also regulated by the level of cholesterol content in the plasma membrane. For example, cholesterol enrichment resulted in strong suppression of capsaicin-induced whole cell TRPV1 currents that was not mimicked by epicholesterol administration [30, 31]. Interestingly, TRPV4 also contains cholesterol recognition motifs within it TM4-Loop4-TM5 region [17]. This suggests the possibility that membrane cholesterol directly interacts with the channel possibly preventing its ability to be activated by muscarinic receptor signaling. Alternatively, changes in membrane cholesterol may alter its positioning within the membrane relative to muscarinic receptors.

In contrast to what we observed for muscarinic receptor activation, direct activation of TRPV4 elicits endothelium-dependent dilation in arteries from both normoxic and CH rats. In normoxic animals, activation of TRPV4 channels with GSK101 elicits an SK_ca_/IK_ca_-dependent dilation, suggesting that Ca^2+^ influx through TRPV4 channels can activate Ca^2+^-activated K^+^ channels in endothelial cells in a manner analogous to the relationship between ryanodine channel-mediated Ca^2+^ sparks and BK_ca_ channels in vascular smooth muscle. This is consistent with studies that have linked TRPV4 sparklets to SK_ca_/IK_ca_ channel activation [39]. However, in arteries from CH rats, inhibition of all three K_ca_ channels was required to inhibit GSK101-induced dilation. The data suggest that all three channels are active following CH, and that in the absence of SK and IK channels, BK channels are able to regulate endothelial cell membrane potential and elicit maximal vasodilation. Conversely, in the presence of iberiotoxin, SK and IK channels regulate membrane potential as they do in control arteries. We have previously investigated the K_ca_ channel subtypes involved in ACh-induced dilation in arteries from control and CH rats in the presence of L-NNA and indomethacin [13]. In this experiment, EDH-dependent dilation in response to ACH administration was abolished by SK/IK inhibition in arteries from control animals, luminal iberiotoxin administration was without effect. In arteries from CH rats, SK_ca_/IK_ca_ inhibition tended to attenuate the ACh response. However, luminal iberiotoxin treatment alone was able to abolish ACh-induced dilation in these arteries. These results suggest a switch between ACh-dependent activation of SK/IK channels to an entirely eBK channel-mediated dilation following CH. The discrepancy between the channels mediating ACh- and GSK101-induced dilation in arteries from CH rats could be due to differences in agonists used in the two studies. Direct pharmacological activation of TRPV4 may result in a large Ca^2+^ influx through TRPV4 channels capable of activating all three K_ca_ channel subtypes. Whereas, muscarinic receptor signaling activates more spatially discrete TRPV4 Ca^2+^ events.

There is a well-established belief that endothelial cells do not express BK channels and/or that they do not participate in vascular control. However, our previous work as well as the current study provides evidence that endothelial cells contain functional BK channels following CH [13, 33, 34]. We have shown expression of endothelial BK channels in the endothelium of gracilis arteries using immunofluorescence [33]. In addition, functional experiments have demonstrated an effect of luminal administration of iberiotoxin to restore constrictor reactivity, prevent ACH-induced dilation in arteries from CH rats, as well as block ACh-induced endothelial cell membrane potential hyperpolarization [13]. Indeed, endothelial cells from aorta and gracilis arteries isolated from rats exposed to CH exhibit BK currents [13, 33, 34] which are absent in cells from normoxic animals. These electrophysiology studies demonstrate that these currents are iberiotoxin-sensitive outward currents and have a unitary conductance (~ 225 pS in symmetrical K^+^) that is in line with the established BK channel conductance [33]. The absence of functional BK channels in endothelial cells from control arteries is due to tonic inhibition of the channel by membrane cholesterol. Indeed, our work has shown that unmasking of functional eBk channels in CH is mediated by a reduction in endothelial membrane cholesterol [33]. Indeed, regulation of BK channel activity by membrane cholesterol has been shown in several studies [reviewed in [2]]. Consistently, the results of the present study demonstrate that endothelial membrane cholesterol is reduced after 48 h of CH supporting the postulate that the appearance of functional endothelial BK channels following CH may be mediated by a reduction in endothelial membrane cholesterol. This inhibitory effect appears to be mediated by a protein-sterol interaction rather than to changes in membrane fluidity, since substitution of membrane cholesterol with either epicholesterol or ent-cholesterol has no effect on BK channel activity [1].

Administration of ACh increased Ca^2+^ events within the endothelium of arteries from both control and CH rats. Calcium influx through TRPV4 channels presumably activates endothelial K_ca_ channels. However, it is unclear if TRPV4-dependent Ca^2+^ influx directly activates K_ca_ channels or acts indirectly through a secondary release of Ca^2+^ from the endoplasmic reticulum through IP_3_ receptors. Indeed, in mouse vertebral artery smooth muscle cells, TRPV4-mediated activation of BK channels involves a secondary Ca^2+^ release event through ryanodine receptors rather than direct Ca^2+^ sparklet activation of the channel [4]. In contrast, there is evidence that supports the postulate that Ca^2+^ influx through TRPV4 channels is sufficient to activate eBK_ca_ channels. Indeed, a cluster of TRPV4 channels have been predicted using mathematical models to act cooperatively to elicit micromolar increases in Ca^2+^ within a few square micrometers of K_ca_ channels [29]. This prediction of cooperative gating of clusters of TRPV4 channels has been demonstrated in mouse mesenteric arteries [39]. Moreover, we have previously shown that endothelial BK channels have greater sensitivity to Ca^2+^ compared to BK channel on vascular smooth muscle [33]. These studies demonstrated an increase in channel open probability at sub-micromolar Ca^2+^ concentrations. Taken together, this evidence suggests that Ca^2+^ influx through TRPV4 would be sufficient to activate endothelial BK channels.

Our initial observations demonstrating blunted vasoconstrictor reactivity following CH were performed in conscious control and CH rats [3]. Vasoconstrictor responses were examined in these animals under both room air and acute hypoxic conditions. We found that CH-induced reduction in vasoconstriction remained even after return to room air conditions and that the blunted responses were exaggerated if the experiment was performed during exposure to acute hypoxia (i.e., superimposed hypoxic dilation response). The interplay among membrane cholesterol, eBK, and TRPV4 channels in mediating acute hypoxic vasodilation has not been investigated. Given the rapidity with which hypoxic dilation occurs (i.e., minutes), it seems unlikely that this response could involve alteration in membrane cholesterol content and hence eBK channel activation. Although we have not established the mechanism of CH-induced reduction in membrane cholesterol (e.g., altered trafficking or de novo synthesis), it is unlikely that acute exposure (minutes) would alter membrane cholesterol content that would unmask eBK and TRPV4 channels in a manner similar to CH.

In summary, our results demonstrate that functional TRPV4 channels are expressed on gracilis artery endothelial cells, but are not involved in EDH-dependent dilation in normoxic animals. However, in arteries from CH animals, TRPV4 channels participate in EDH-mediated dilation. Ca^2+^ influx through these channels appears to participate in activation of all three K_ca_ channel subtypes following CH. Taken together, these results provide additional support for the existence of function BK_ca_ channels in the endothelium, which may be regulated in a manner similar to SK_ca_ and IK_ca_ channels.

## Limitations

This series of studies relies on pharmacological inhibition of TRPV4 using small molecule antagonists to elucidate the mechanism of eBK channel activation following CH. Although large differences in potency have been observed for the TRPV4 antagonist used in this study compared with other TRP channel family members, it is possible that these compounds also inhibit other TRP channel members given the degree of protein sequence homology among subfamily members [24]. Futures studies employing the use of endothelial specific TRPV4 knockout animals will help resolve this issue.

## Future directions

As mentioned above, there is preliminary evidence to suggest that TRPV channel family members may be regulated by membrane cholesterol. It is conceivable that the lack of a role of TRPV4 in ACh-induced dilation in control animals is mediated by a protein-sterol interaction between TRPV4 and membrane cholesterol that renders it unavailable to downstream muscarinic receptor signaling. Studies are needed to investigate the role of membrane cholesterol in the regulation of TRPV4 channel activity. Additional studies are also needed to determine the mechanism by which CH decreases membrane cholesterol. Moreover, the concept that membrane cholesterol content can result in changes in vascular function needs further study. Indeed, if hypercholesterolemia results in an enhancement of endothelial plasma membrane cholesterol content which secondarily impairs vascular function is unknown.

## Abbreviations

CH: Chronic hypoxia
eBK: Endothelial large conductance Ca^2+^-activated K^+^ channel
EDH: Endothelial-dependent hyperpolarization
GSK101: GSK101670A
IbTx: Iberiotoxin
IK_ca_: Intermediate conductance Ca^2+^-activated K^+^ channel
L-NNA: N-nitro-L-arginine
MβCD: Methyl-β-cyclodextrin
RAEC: Rat aortic endothelial cells
SK_ca_: Small conductance Ca^2+^-activated K^+^ channel
TRPV4: Transient receptor potential cation channel V4
